# Synthesis and “*in Vitro*” Trypanocidal Activity Evaluation of Some Organo-iron Compounds.

**DOI:** 10.1155/MBD.2002.329

**Published:** 2002

**Authors:** Máfircio L. A. e Silva, Alberto F. Neto, Silvia A. Cardoso, Sérgio Albuquerque, Joseph Miller

**Affiliations:** 1 LABQUIM-Núcleo de Pesquisas em Ciências Exatas e Tecnológicas da Universidade de Franca Av. Armando Salles Oliveira, 201 Franca São Paulo Z.P. 14404-600 Brazil; 2 Dept. de Química da Faculdade de Filosofia Ciências e Letras de Ribeirão Preto USP, Ribeirão Preto Av. Bandeirantes s/n Ribeirão Preto São Paulo Z.P. 14040-903 Brazil; 3 Dept. de Ciências Farmacêuticas da Faculdade de Ciências Farmacêuticas de Ribeirão Preto USP, Ribeirão Preto Av. do Caé s/n Ribeirão Preto São Paulo Z.P. 14040-903 Brazil; 4 Dept. de Ciências da Saúde da Faculdade de Cincias Farmacêuticas de Ribeirão Preto USP, Ribeirão Preto Av. do Café s/n Ribeirão Preto São Paulo Z.P. 14040-903 Brazil; 5 Universidade Federal da Paraíba Laboratório de Tecnologia Farmacêutica P.O. Box-5009 João Pessoa PB 58051-970 Brazil

## Abstract

Eight organo-iron ferrocene derivatives and arenocenium salts were prepared and evaluated by “*in vitro*” assay against one strain of *Trypanosoma cruzi* (Y). Six of the eight organo-iron compounds assayed, piperazinium diferrocenoate **1**, η^6^-(o-xylene)-η^5^-(cyclopentadienyl) Iron(II) hexafluorophosphate **3**, η^6^-(mesitylene)-η^5^-(cyclopentadienyl) iron(II) hexafluorphosphate **5**, η^6^-(durene)-η^5^-(cyclopentadienyl) iron(II) hexafluorphosphate **6**, η^6^-(ρ-chlorotoluene)-η^5^-(cyclopentadienyl) Iron(II) hexafluorphosphate **7**  and η^6^-(chlorobenzene)-η^5^-(cyclopentadienyl) iron(II) picrate **8** , were poorly active in the “*in vitro*” assays. Only two compounds 1,1'–(N-pyperidinocarbonyl) ferrocene **2**(IC_50_=2.4 μg/mL) and η^6^-(o-xylene)-η^5^(cyclopentadienyl) iron(II) picrate **4** (IC_50_=12.08 μg/mL), were more active. Thus, some of the compounds are promising to be used against Chagas' disease as a prophylactic agents.


### SYNTHESIS AND "*IN VITRO*" TRYPANOCIDAL ACTIVITY EVALUATION OF SOME ORGANO-IRON COMPOUNDS.

Márcio L. A. e Silva*[1,2], Alberto F. Neto[3], Silvia A. Cardoso[3], Sérgio Albuquerque[4] and Joseph Miller[5]

[1] LABQUIM-Núcleo de Pesquisas em Ciências Exatas e Tecnológicas da Universidade de Franca, Av. Armando Salles Oliveira, 201, Z.P. 14404-600, Franca, São Paulo, Brazil

[2] Dept. de Química da Faculdade de Filosofia, Ciências e Letras de Ribeirão Preto , USP, Ribeirão Preto, Av. Bandeirantes s/n, Z.P. 14040-903, Ribeirão Preto, São Paulo, Brazil

[3] Dept. de Ciências Farmacêuticas da Faculdade de Ciências Farmacêuticas de Ribeirão Preto, USP, Ribeirão Preto, Av. do Café s/n, Z.P. 14040-903, Ribeirão Preto, São Paulo, Brazil

[4] Dept. de Ciências da Saúde da Faculdade de Ciências Farmacêuticas de Ribeirão Preto, USP, Ribeirão Preto, Av. do Café s/n, Z.P. 14040-903, Ribeirão Preto, São Paulo, Brazil

[5] Universidade Federal da Paraíba, Laboratório de Tecnologia Farmacêutica, P.O. Box-5009, 58051-970, João Pessoa , PB, Brazil

**ABSTRACT**

Eight organo-iron ferrocene derivatives and arenocenium salts were prepared and evaluated by "*in vitro*" assay against one strain of *Trypanosoma cruzi* (Y). Six of the eight organo-iron compounds assayed, piperazinium diferrocenoate **1**, $\eta^6$-(*o*-xylene)-$\eta^5$-(cyclopentadienyl) Iron(II) hexafluorophosphate **3**, $\eta^6$-(mesitylene)-$\eta^5$-(cyclopentadienyl) iron(II) hexafluorphosphate **5**, $\eta^6$-(durene)-$\eta^5$-(cyclopentadienyl) iron(II) hexafluorphosphate **6**, $\eta^6$-(ρ-chlorotoluene)-$\eta^5$-(cyclopentadienyl) Iron(II) hexafluorphosphate **7** and $\eta^6$-(chlorobenzene)-$\eta^5$-(cyclopentadienyl) iron(II) picrate **8**, were poorly active in the "*in vitro*" assays. Only two compounds 1,1'-(N-pyperidinocarbonyl) ferrocene **2** ($IC_{50}$=2.4 μg/mL) and $\eta^6$-(*o*-xylene)-$\eta^5$-(cyclopentadienyl) iron(II) picrate **4** ($IC_{50}$=12.08 μg/mL), were more active. Thus, some of the compounds are promising to be used against Chagas' disease as a prophylactic agents.

### INTRODUCTION

The therapeutic use of metals or inorganics salts is secular[1]. Paracelso (in 1700), preconized the use of the minerals and iron, cadmium, mercury, arsenic, gold and antimony salts as medicine ("Iatrochemistry") in oposite to the vitalist hypothesis ("Vital Force") accepted at that time.

The rational use of metals, semi-metals and their compounds in therapeutic started with Lissauer in 1865 who administered a topic antiseptic, an arsenical formulation called "Fowler Licor", to the leucemics patients[2]. Therefore, the use of metals in the therapeutics had a greatest increase, as an example, the use of cacodilates and cacodil oxides (alquil arseniates) and injections of coloidals arsenic, silver, gold and antimony in oncology chemotherapy[2].

Relegated to a second plane, the new metal drug researchs practically stopped until the discovery of the use of cisdiaminedichoroplatinum(II) complex (***Cisplatin®***) with bactericidal, cytostatic and antineoplastic properties, by Rosenberg in 1969[1-3].

Among the few developed potencial organo-iron drugs[1,2], in recent years ferrocene based anti-malarial agents have been described and the ferrocene analogs of chloroquine, mefloquine and quinine have been tested[4-6].

Silva *et al.* in 1999[7], reported a significative analgesic effect produced by $\eta^6$-(anisole) triscarbonyl chromium(0), a chromium derivative complex.

Chagas' disease was characterized in the Americas in 1909[8]. Since this time, not much progress has been achieved for controlling this serious illness. This is in part because of the high interaction between the parasite and its hosts. Today, fewer drugs are used against Chagas' disease. Therefore, these drugs were showed to be efficient over time.

The aim of this work was the synthesis of some organo-iron compounds and their trypanocidal evaluation, attempting to discover a new drug class against Chagas' disease.

### MATERIALS AND METHODS

### Experimental

Piperazinium diferrocenoate **1** and 1,1'-(N-piperidinocarbonyl)ferrocene **2**, were prepared as previously published[9].

Fully experimental details, apparatus and assembly for the improved ligand exchange reaction in ferrocene, under microwave irradiation conditions, were recently submitted for publication[10]. All the arenocenium salts used in this work: $\eta^6$-(*o*-xylene)-$\eta^5$-(cyclopentadienyl) iron(II) hexafluorophosphate **3**, $\eta^6$-(*o*-xylene)-$\eta^5$-(cyclopentadienyl) Iron(II) picrate **4**, $\eta^6$-(mesitylene)-$\eta^5$-(cyclopentadienyl) iron(II) hexafluorphosphate **5**, $\eta^6$-(durene)-$\eta^5$-(cyclopentadienyl) Iron(II) hexafluorophosphate **6**, $\eta^6$-(p-chlorotoluene)-$\eta^5$-(cyclopentadienyl) iron(II) hexafluorophosphate **7** and $\eta^6$-(chlorobenzene)-$\eta^5$-(cyclopentadienyl) iron(II) picrate **8** were prepared by a further modification[13] of this procedure. We found that the aluminum metal powder may be substituted by zinc powder with equivalent results and a teflon beaker may be used, instead of the Berzelius flask (tall beaker), avoiding any risk of glassware breakage.

**Strain of *Trypanosoma cruzi***

The compounds were tested against the strain Y[11] of *T.cruzi.* The Y strain shows predominantly slender blood trypomastigote forms with macrophage tropism and is characterized in phylogenetic lineage I and the *Trypanosoma cruzi* II group.

**In vitro Bioassay**

The bioassays were carried out using blood of infected Swiss albino mice, collected by cardiac puncture at the peak of the parasitemic infection (7$^{th}$ day of infection for Y strain). The infected blood was diluted to achieve a concentration of $10^6$ trypomastigote forms/mL. The solutions of the compounds were prepared in DMSO and were added into the infected mouse blood to provide concentrations of 100, 250 and 500 µm/mL, respectively. The bioassays were performed in triplicate on microtiter plates (96 walls), which contained 200 µL of mixture for wall. The plates were incubated at 4 °C for 24 hours under constant stirring. Afterwards, the trypanocidal activity was evaluated by counting the trypomastigote forms of the parasite remaining, following the method described by Brener[12]. Controls containing either DMSO or gentian violet at 250 µg/mL were processed in parallel.

### RESULTS AND DISCUSSION

The relative low toxicity of the ferrocene group[13], and their well established aromatic character[13], similar to the benzene group, as well the electron aceptor properties of the metal ligand moieties[7], makes ferrocene and other metal-ligand groups attractive for the use in drug design.

We prepared some organo-iron compounds, ferrocene derivatives (**1** and **2**) and arenocenium salts (**3-8**) (**Figure 1**) and screened then for tripanocidal activity.

**FIGURE 1.** CHEMICAL STRUCTURES OF THE COMPOUNDS TESTED

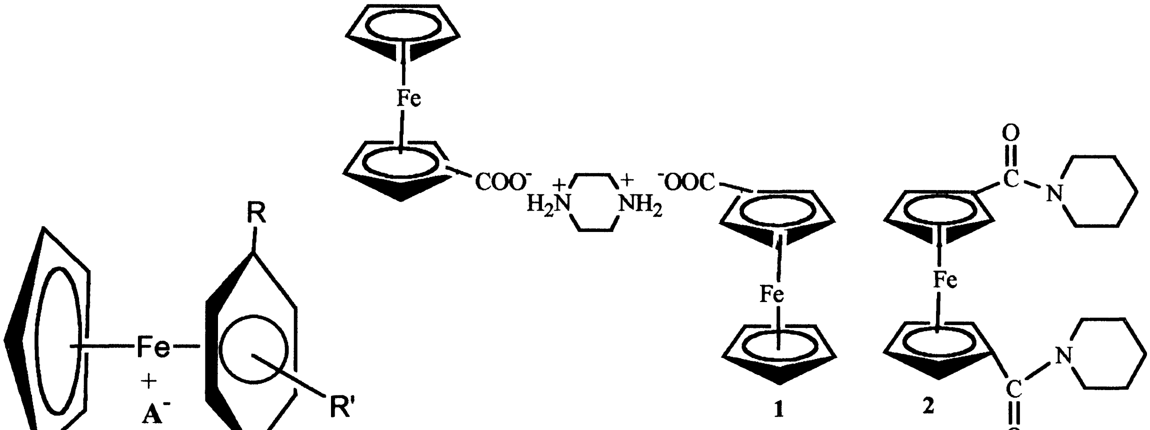

**3** ortho xylene (R=methyl; R'=2-methyl) $PF_6^-$

**4** ortho xylene (R=methyl; R'=2-methyl) picrate

**5** mesitylene (R=methyl; R'=3,5-dimethyl) $PF_6^-$

**6** durene (R=methyl; R'=2,3,4-dimethyl) $PF_6^-$

**7** *p*-chlorotoluene (R=methyl; R'=Cl)

**8** chlorobenzene (R=H; R'=Cl) $PF_6^-$

A = $PF_6$, Picrate

The arenocenium salts were prepared by a modification of the recently submitted[10] procedure for the ligand exchange reaction of ferrocene and arenes, induced by microwave irradiation.

During the application of this procedure to the synthesis of the target compounds used in this work, we discovered that the anti-economical excess of ferrocene[15], used in the original procedure of Dabirmanesh *et al.*[15] is not necessary, and a large excess of the aluminum trichloride as catalyst increase the yields, similarly to that we observed[15] with the old thermal induced procedure for ferrocene ligand exchange.

Also, Astruc *et al.*[16] reported that the ligand exchange was a non-quantitative reaction, and large amounts of unreacted ferrocene remain unchanged. We verified that in microwave conditions, the ligand exchange reaction is quantitative with respect of the ferrocene amount. In the original procedure, Dabirmanesh *et al.*[15] could not observed the same results, because they carried out the reactions ever in the presence of large excess of ferrocene.

It seems to us that due to the high capacity of ionization of the microwaves, which may increase the speed of many chemical reactions[15], in these conditions, ferrocene can be very easily cleaved for the efficient formation of the cyclopentadienyl iron(II) cation[17,18], the reactive intermediate of the ligand exchange reaction.

**Table I** shows the results of *T. cruzi* biological assays. We can observe that all compounds evaluated did not present very high activity against the parasite. However, we can see a high therapeutic potential, by the significant results found in the evaluation, in the compounds **2** and **4**, that shown low $IC_{50}$ values (2,4 and 12,08 μg/mL, respectively). In spite of these compounds not showing a high activity against the parasites (57 % and 69 % of lysis), the results are very interesting, mainly because they are practically insoluble in the protocol used for the trypanocidal activity evaluation.

**TABLE I.** IN VITRO[a,b] TRYPANOCIDAL Of ORGANO-IRON COMPOUNDS AGAINST Y STRAIN Of *T. cruzi.*

| Compounds | Trypanocidal activity (lise % ±SD) Dose (μg/mL)-Y strain | | | $IC_{50}$ μg/mL |
|---|---|---|---|---|
| | 100 | 250 | 500 | |
| **1** | 44.0 ± 3 | 69.0 ± 0 | 79.0 ± 3 | 124.0 |
| **2** | 55.0 ± 6 | 55.0 ± 5 | 57.0 ± 9 | 2.40 |
| **3** | 29.0 ± 4 | 63.4 ± 2 | 73.3 ± 4 | 189.3 |
| **4** | 61.3 ± 3 | 65.0 ± 5 | 69.0 ± 7 | 12.08 |
| **5** | 50.0 ± 8 | 61.0 ± 5 | 62.0± 6 | 89.95 |
| **6** | 44.5 ± 5 | 49.0 ± 5 | 67.0 ± 3 | 80.08 |
| **7** | 48.2 ± 2 | 59.0 ± 3 | 65.4 ± 3 | 116.8 |
| **8** | 28.3 ± 3 | 56.0 ± 0 | 59.2 ± 3 | 257.6 |

[a] Positive control, gentian violet, concentration of 250 μg/mL (100 % lysis) – $IC_{50}$ = 76 μg/mL.
[b] Negative controls. Mice infected blood without any added compound and mice blood infected containing the same DMSO concentration used in the stock solutions, have not showed any reduction of the parasite numbers.

We should point out the polymorphism existent among the trypomastigote forms of the same lineage of *T.cruzi* that can present a difference in the susceptibility of the parasite in relation to the evaluated compounds. We do not have an explanation for this, but it's known that the evolution of *T.cruzi* depends on several biological and genetic factors, inherent to the development of the parasite, hindering the Chagas' disease therapeutics.

Although the partial effectiveness for the trypanocidal activity, it opens a pathway for the synthesis of other new derivatives and we may could evaluate the correlation between the chemical structure and the type of biological activity, characteristic for this class of compounds.

**ACKNOWLEDGEMENTS**

We acknowledge to CAPES (Coordenadoria de Aperfeiçoamneto de Pessoal de Nível Superior) for a bursary to Márcio Luís Andrade e Silva and to the Fundação de Amparo à Pesquisa do Estado de São Paulo – FAPESP, for the financial support of this work.

**REFERENCES**
1- P. Köpf-Maier, *Eur. J. Clin. Pharmacol.*, **47**, 1 (1994).
2- I. Haiduc, C. Silvestru, "*Organometallics in Cancer Chemotherapy*", CRC Press., vol. 2 (1989).
3- B. Rosenberg, L. Van Camp, J.E. Trasko, V.H. Mansour, *Nature*, **222**, 385 (1969).
4- B. Pradines, A. Tall, C. Rogier, A. Spiegel, J. Mosnier L. Marrama; T. Fusai, P. Millet, E. Panconi, J.F. Trape, D. Parzy, *Trop. Med. Int. Health*, **7**, 265 (2002).
5- B. Pradines, T. Fusai, W. Daries, V. Laloge, C. Rogier, P. Millet, E. Panconi, M. Kombila, D. Parzy, *J. Ant. Chem.*, **48**, 179 (2001).
6- C. Biot, L. Delhaes, L.A. Maciejewski, M. Mortuaire, D. Camus, D. Dive, J.S. Brocard, *Eur. J. Med. Chem.*, **35** 707 (2000).
7- M.L.A. Silva, A. Federman Neto and J. Miller, *Metal-Based Drugs*, **6**, 25 (1999).
8- C. Chagas, Mem. Inst. Oswaldo Cruz, **1**, 159-219 (1919) In: J.K. Bastos, S. Albuquerque and M.L.A. Silva, *Planta médica*, **65**, 541 (1999).
9- A. Federman Neto, J. Miller, V. F. Andrade, S. Y. Fujimoto, M. M. F. Afonso, F. C. Archanjo, V. A. Darin, M. L. A. Silva, A. D. L. Borges, G. Del Ponte, *Z. Anorg. Allg. Chem*., **628**, 209 (2002).
10- A. Federman Neto, P.L. A.G Cordo, M. L. A. Silva, *Quim. Nova,* (2002). *In Press*
11- L.H. Pereira da Silva, V. Nussenzweig, *Fol. Clín. Biol.*, **20**, 191 (1953).
12- Z. Brener, *Rev. Inst. Med. Trop.*, **4**, 389 (1962).
13- L.G. Valerio, D.R. Petersen, *Exp. Mol. Path.*, **68**(1), 1 (2000).
14- M. Laskoski, W. Steffen, M.D. Smith, U.H.F. Bunz, *J. Chem Soc. Chem Comm.,* **8**, 691 (2001).
15- Q. Dabirmanesh, S. I. S. Fernando, R. M. G. Roberts, *J. Chem. Soc. Perkin Trans.* **1**, 743 (1995).
16- A. Federman Neto, J. Miller, *An. Acad. Bras. Ciênc.*, ***54***, 332 (1982).
17- D. Astruc, R. Dabard, *J. Organomet. Chem.* , **111**, 339 (1976).
18- T. Hayashi, Y. Okada, S. Shimizu, *Trans. Met. Chem.*, **21**, 418 (1996).

**Received: May 8, 2002 – Accepted: May 16, 2002 –**

**Accepted in publishable format: May 21, 2002**

